# Managing chronic lymphocytic leukemia in 2020: an update on recent clinical advances with a focus on BTK and BCL-2 inhibitors

**DOI:** 10.12703/r/10-22

**Published:** 2021-02-26

**Authors:** Prithviraj Bose, Varsha Gandhi

**Affiliations:** 1 Department of Leukemia, University of Texas MD Anderson Cancer Center, Houston, TX 77030, USA; 2 Department of Experimental Therapeutics, University of Texas MD Anderson Cancer Center, Houston, TX 77030, USA

**Keywords:** Chronic lymphocytic leukemia, ibrutinib, venetoclax, reversible BTK inhibitors, acalabrutinib, zanubrutinib, obinutuzumab, duvelisib

## Abstract

The therapeutic landscape of chronic lymphocytic leukemia (CLL) underwent a paradigm shift in 2014 with the approval of ibrutinib, which binds covalently to the C481 residue of Bruton’s tyrosine kinase (BTK) and irreversibly inhibits it. A number of large, phase 3 trials conducted in both the frontline and the relapsed/refractory settings resulted in the approval of ibrutinib for all CLL. Indeed, the role of chemoimmunotherapy in CLL is fast dwindling. The limitations of ibrutinib, e.g. the development of resistance-conferring C481 BTK mutations and the toxicity issues of atrial fibrillation and bleeding, in particular, have also become apparent with longer-term follow-up. This has spurred the development of second-generation, irreversible inhibitors with greater selectivity for BTK and third-generation, reversible BTK inhibitors to address C481 site mutations. The last 3 years have also witnessed enormous growth in the therapeutic role of the B-cell lymphoma 2 (BCL-2) antagonist venetoclax, initially approved (in 2016) only for patients with relapsed, 17p-deleted CLL. Venetoclax, in combination with CD20 antibodies, is currently approved for both treatment-naïve and relapsed/refractory patients, regardless of genomic subtype. Robust results have also been reported for ibrutinib plus venetoclax, and “triple” combinations of a BTK inhibitor, venetoclax, and obinutuzumab are now being pursued. The major questions facing the field at present are how best to select patients for BTK inhibitor monotherapy versus venetoclax/obinutuzumab upfront, what to do after failure of both BTK inhibitor(s) and venetoclax, and the ideal way to integrate measurable residual disease data into decisions regarding treatment choice, duration, and discontinuation.

## Introduction

The emergence of an array of highly effective targeted therapies that exploit several intrinsic vulnerabilities of chronic lymphocytic leukemia (CLL) cells, particularly small molecules that target Bruton’s tyrosine kinase (BTK) to interfere with B-cell receptor (BCR) signaling or the anti-apoptotic function of B-cell lymphoma (BCL-2), has transformed the treatment landscape of this disease and made it one of the most gratifying hematologic malignancies to treat. With these unprecedented successes have come new challenges: the costs and unique adverse events (AEs) associated with indefinite BTK blockade, the problem of relapse after “time-limited” (1–2 year) venetoclax/anti-CD20 monoclonal antibody regimens, the immune-mediated AEs observed with the phosphatidylinositol-3-kinase (PI3K) inhibitors, and the risk of tumor lysis syndrome (TLS) with venetoclax-based regimens, etc. The best frontline regimen for patients with CLL continues to evolve, and the optimal sequencing of therapies in later lines remains unclear. Treatment of Richter’s transformation (RT) remains unsatisfactory and a major unmet clinical need. In this article, we update our last review of the topic^1^, covering the major and practice-changing therapeutic advances in CLL over the last 3 years with a focus on the BTK inhibitors and venetoclax. A comprehensive review on the treatment of CLL was recently published^2^.

## Update on ibrutinib monotherapy

After ibrutinib’s Food and Drug Administration (FDA) approval as monotherapy for both relapsed/refractory and treatment-naïve patients with CLL based on the RESONATE^3^ and RESONATE-2^4^ phase 3 randomized controlled trials (RCTs), the results of several important phase 3 trials comparing ibrutinib-based regimens to chemoimmunotherapy (CIT) have been reported in the last 3 years, firmly establishing ibrutinib in the frontline setting for both younger and older patients. In the final analysis of RESONATE with a median follow-up of 65.3 months, median progression-free survival (PFS) remained significantly longer for patients randomized to ibrutinib versus ofatumumab (44.1 versus 8.1 months, *P* <0.001)^5^. These results were virtually identical in the genomically high-risk subset of patients (deletion 17p [del17p], *TP53* mutation [*TP53^mut^*], del11q, unmutated *IGHV*), who comprised 82% of the study population. The overall response rate (ORR) to ibrutinib was 91%, with 11% of patients achieving complete response (CR) or CR with incomplete count recovery (CRi). Hypertension and atrial fibrillation occurred in 21% and 12% of patients, respectively (grade ≥3 in 9% and 6%), with 16% of patients discontinuing ibrutinib because of AEs. Both the PFS and overall survival (OS) benefits of ibrutinib over chlorambucil were sustained after a median follow-up time of 60 months in the RESONATE-2 trial^6^. The 5-year PFS and OS estimates were 70% and 83% for ibrutinib and 12% and 68% for chlorambucil, respectively. The investigator-assessed ORR to ibrutinib was 92% with a CR rate of 30% in this frontline trial. In the context of ibrutinib monotherapy, use in the frontline setting is associated with a higher likelihood of CR, as is the lack of bulky lymphadenopathy (≥5 cm)^7^. While it remains unclear whether achievement of CR with ibrutinib impacts long-term outcomes, a study from the MD Anderson Cancer Center (MDACC) reported a statistically significant association between CR and PFS^8^. The rate of 2-year PFS was 85% in an investigator-initiated trial conducted at the National Institutes of Health (NIH) in 34 previously untreated CLL patients with *TP53* alterations who received ibrutinib monotherapy; median time to progression (TTP) was 53 months^9^.

## Ibrutinib versus CIT

The results of two important US Intergroup phase 3 studies comparing ibrutinib-based regimens in the frontline setting against standard CIT regimens in both older and younger patients with CLL were recently published. The Alliance trial (A041202) randomized 547 older patients (≥65 years of age) with previously untreated CLL to receive one of ibrutinib alone, ibrutinib plus rituximab, or bendamustine plus rituximab (BR)^10^. At the time of publication, median PFS had not been reached in either of the ibrutinib-containing arms. Importantly, ibrutinib was continued until disease progression, while BR was administered for a standard 6 cycles. The estimated 2-year PFS rate was 74% for BR, 87% for ibrutinib alone, and 88% for ibrutinib/rituximab. PFS was not significantly different between the two ibrutinib-containing groups. No differences were apparent among the three arms in terms of OS at a median follow-up of 38 months. The rate of grade ≥3 hematologic AEs was higher in the BR arm (61%) than in the ibrutinib-containing arms (≈40%), but that of grade ≥3 non-hematologic AEs was higher in each ibrutinib-containing arm (74%) than in the BR arm (63%).

The Eastern Cooperative Oncology Group (ECOG) 1912 trial randomized 529 treatment-naïve patients with CLL ≤70 years of age 2:1 to receive ibrutinib (until disease progression) plus rituximab (6 cycles) or 6 cycles of fludarabine, cyclophosphamide, and rituximab (FCR)^11^. Patients with del17p CLL were excluded from this trial given their known poor outcomes with CIT. After a median of 33.6 months of follow-up, this trial demonstrated both a PFS and an OS advantage for ibrutinib plus rituximab over FCR (3-year PFS, 89.4% versus 72.9%; 3-year OS, 98.8% versus 91.5%, *P* <0.001 for both comparisons). Importantly, there was no difference in 3-year PFS (87.7% for ibrutinib/rituximab and 88% for FCR) between the two arms when considering only the *IGHV*-mutated patients, in line with the known excellent long-term outcomes with FCR in this subgroup^12^. An update of the ECOG trial with longer follow-up (median 45 months) was presented at the 2019 American Society of Hematology (ASH) annual meeting^13^. The rate of grade ≥3 AEs was 70% in the ibrutinib/rituximab arm and 80% in the FCR arm (*P* = 0.013). A total of 95 patients discontinued ibrutinib (24% owing to disease progression or death, 51% owing to AEs or complications, and 25% owing to withdrawal of consent or other reasons), after which the median time to disease progression or death was 23 months. Only an increased baseline Cumulative Illness Rating Scale (CIRS) score predicted discontinuation of ibrutinib for reasons other than disease progression or death.

## Ibrutinib addition to CIT

An interesting strategy being pursued at MDACC to optimize FCR for younger patients with *IGHV*-mutated CLL and no del17p/TP53^mut^ is the combination of ibrutinib, fludarabine, cyclophosphamide, and obinutuzumab (iFCG), in which the chemotherapy is limited to 3 courses in an effort to reduce the risk of therapy-related myeloid neoplasms (t-MNs) and the duration of ibrutinib and obinutuzumab is determined through assessments of measurable residual disease (MRD) at different time points. Recently presented results from this study showed that the best ORR was 98% (44 of 45 patients) and the rates of bone marrow MRD clearance were 87% after 3 cycles and 89% after 6 cycles^14^. A total of 41 patients reached the 1-year time point and all discontinued therapy, being MRD negative in the bone marrow; no patient had experienced clinical relapse after a median follow-up of 22.7 months since ibrutinib discontinuation. There was one MRD recurrence and one patient developed therapy-related myelodysplastic syndrome at a median follow-up of 34.2 months.

Investigators at the Dana Farber Cancer Institute (DFCI) have studied ibrutinib (for up to 2 years) in combination with 6 cycles of FCR (iFCR) in unselected, younger (≤65 years of age) patients with CLL: of 85 patients enrolled, 71 (84%) achieved a best response of undetectable MRD in the bone marrow, while a third of patients achieved CR plus undetectable bone marrow MRD 2 months after the last cycle of iFCR^15^.

Although the addition of rituximab to ibrutinib does not extend PFS beyond that achieved with ibrutinib alone^10,16^, the same may not be true of obinutuzumab, a type II glycoengineered CD20 monoclonal antibody proven superior to rituximab in the pivotal German CLL11 trial^17^. Obinutuzumab possesses enhanced antibody-dependent cellular cytotoxicity (ADCC) compared to rituximab, a process that may, at least preclinically, be antagonized by ibrutinib via inhibition of interleukin-2-inducible T-cell kinase (ITK)^18^. While there has been no direct comparison of ibrutinib plus obinutuzumab against ibrutinib alone, the former regimen is now FDA approved for frontline therapy based on the industry-sponsored iLLUMINATE trial^19^. In this phase 3 RCT, 229 patients with previously untreated CLL aged ≥65 years or younger than 65 with coexisting conditions were randomized 1:1 to receive 6 cycles of obinutuzumab and either continuous daily ibrutinib (until disease progression) or chlorambucil (0.5 mg/kg on days 1 and 15 every 28 days for 6 cycles). After a median follow-up of 31.3 months, the median PFS in the ibrutinib/obinutuzumab group had not been reached, while that in the chlorambucil/obinutuzumab group was 19 months (*P* <0.0001). The estimated 30-month PFS rates were 79% and 31%, respectively. Serious AEs occurred in 58% of patients in the ibrutinib/obinutuzumab group and in 35% of patients receiving chlorambucil/obinutuzumab.

## Ibrutinib toxicities and the need for more selective BTK inhibitors

Overall, ibrutinib is well tolerated; in an integrated safety analysis of ibrutinib-treated (for up to 43 months) patients from RESONATE (n = 195) and RESONATE-2 (n = 135), the most frequent AEs were diarrhea (52%, grade 3 in 5%) and fatigue (36%, grade 3 in 3%)^20^. The most common grade 3/4 AEs were neutropenia (18%) and pneumonia (12%). The prevalence of hypertension increased over time. Dose reductions and discontinuation due to AEs occurred in 13% and 11% of patients, respectively. In a “real world” analysis of 616 patients with a median follow-up of 17 months, an estimated 41% discontinued ibrutinib, mostly because of toxicities, after a median of 7 months^21^. Atrial fibrillation is, of course, a well-established risk associated with ibrutinib therapy^22^. Pooled data on 1,505 CLL and mantle cell lymphoma (MCL) patients participating in 4 RCTs of ibrutinib showed an estimated cumulative incidence of atrial fibrillation of 13.8%; over 85% of patients with atrial fibrillation were able to remain on ibrutinib, and over half received common anticoagulant/antiplatelet medications^23^. In contrast, the risk of ibrutinib-related atrial fibrillation was as high as 38% at 2 years in one “real world” study^24^. Investigators at the Ohio State University (OSU) reported a cumulative incidence of 5.9%, 7.5%, and 10.3% at 0.5, 1, and 2 years, respectively, in a study of 582 ibrutinib-treated patients with a median follow-up of 32 months^25^. Another serious concern with ibrutinib has been the risk of ventricular arrhythmias^26,27^. Indeed, in the three-arm phase 3 Alliance RCT, it was speculated that these might have precipitated a number of otherwise unexplained or unwitnessed deaths on the ibrutinib-containing arms^10^. In an earlier report, 10 cases of sudden death or cardiac arrest were identified in published clinical trials of ibrutinib amongst approximately 1,000 total participants^26^. In the OSU experience, 78.3% of 562 consecutive patients receiving ibrutinib for B-cell malignancies developed new (71.6%) or worsened hypertension, associated with an increased risk of major adverse cardiovascular events, over a median of 30 months^28^. Of note, the cardiovascular effects of ibrutinib have been attributed, at least in part, to off-target inhibition of other kinases^29,30^, suggesting an advantage for more selective inhibitors of BTK in this regard. This is less clear with respect to the bleeding diathesis induced by BTK inhibitors, with conflicting results in studies comparing the effects of ibrutinib and newer, more selective BTK inhibitors on platelet function^31–33^.**

The group at OSU also reported 23 cases of opportunistic infection over 1,225 patient-years of ibrutinib exposure in 566 patients with B-cell malignancies, i.e. an incidence rate of 1.9 per 100 person-years^34^. The majority of these were invasive fungal infections, particularly invasive aspergillosis, as has been reported by others^35^. Preclinically, it has been shown that BTK blockade by ibrutinib impairs phagocytosis of *Aspergillus fumigatus* by macrophages^36^ and that neutrophils in patients receiving ibrutinib also develop multiple functional defects that impair their response against this pathogen^37^.

## Second-generation BTK inhibitors

Similar to ibrutinib, acalabrutinib and zanubrutinib are irreversible inhibitors of BTK that bind covalently to the Cys481 residue. Indeed, point mutations at this binding site, e.g. C481S, have been shown to confer resistance to acalabrutinib^38^, just as they do to ibrutinib^39^. However, these second-generation BTK inhibitors are more selective for BTK than ibrutinib, which might mean improved safety owing to reduced off-target toxicity and could potentially provide more sustained BTK occupancy due to twice daily dosing. Acalabrutinib was approved by the FDA for the treatment of CLL based on the results of 2 phase 3 RCTs, ELEVATE-TN and ASCEND, conducted in the treatment-naïve and relapsed/refractory settings, respectively. The ELEVATE-TN trial compared acalabrutinib alone, acalabrutinib plus obinutuzumab, and chlorambucil plus obinutuzumab in 535 previously untreated patients with CLL ≥65 years of age or younger than 65 but with coexisting conditions^40^. Acalabrutinib’s lack of inhibition of ITK makes it, at least in theory, a better partner for CD20 monoclonal antibodies than ibrutinib and, in a small, single-arm trial conducted at OSU, acalabrutinib plus obinutuzumab produced ORRs of 95% (32% CRs) and 92% (8% CRs) in 19 treatment-naïve and 26 relapsed/refractory patients with CLL, respectively^41^. The 3-year PFS rates were 94% and 88%, respectively. In ELEVATE-TN, acalabrutinib was continued in both arms until disease progression or unacceptable toxicity, while chlorambucil/obinutuzumab was administered for a standard 6 cycles. At a median follow-up of 28.3 months, median PFS had not been reached in either acalabrutinib arm and was 22.6 months for chlorambucil/obinutuzumab (*P* <0.0001 for both comparisons). The 2-year PFS rates were 93% for acalabrutinib/obinutuzumab, 87% for acalabrutinib alone, and 47% for chlorambucil/obinutuzumab. While the trial was not powered for this comparison, an exploratory post hoc analysis showed that acalabrutinib plus obinutuzumab was superior in terms of PFS to acalabrutinib monotherapy. The estimated 2-year OS rates, however, were not significantly different. The ORRs in the acalabrutinib plus obinutuzumab, acalabrutinib monotherapy, and chlorambucil plus obinutuzumab arms were 94%, 86%, and 79%, respectively. The rates of serious AEs were 38.8%, 31.8%, and 21.9%, respectively, and those of grade ≥3 AEs were 70.2%, 49.7%, and 69.8%, respectively. Headache, diarrhea, fatigue, contusion, arthralgia, cough, upper respiratory infection (URI), nausea, dizziness, and neutropenia were all common (≥10%) in the acalabrutinib arms, more so in the acalabrutinib/obinutuzumab arm, although grade ≥3 neutropenia occurred most frequently in the chlorambucil/obinutuzumab arm. Atrial fibrillation occurred in 3.4% of patients receiving acalabrutinib in combination with obinutuzumab and in 3.9% of those receiving acalabrutinib alone.

Final results from the ASCEND study^42^, which compared acalabrutinib monotherapy 1:1 to physician’s choice of either idelalisib plus rituximab (IdR) or BR in 310 patients with relapsed/refractory CLL or small lymphocytic lymphoma (SLL), were recently presented^43^. At a median follow-up of 22 months, median PFS had not been reached for the acalabrutinib arm and was 16.8 months for the BR and IdR arms combined (*P* <0.0001). Overall response rates (including partial response [PR] with lymphocytosis, PR_L_) also favored acalabrutinib (92%, versus 88% for IdR/BR). The 18-month OS rate was 88% for both acalabrutinib and IdR/BR, but 51% of the IdR/BR patients had crossed over to receive acalabrutinib upon disease progression. AEs led to drug discontinuation in 16% of acalabrutinib, 56% of IdR, and 17% of BR patients. The rates of atrial fibrillation, major hemorrhage, and grade ≥3 infections were 6% and 3%, 3% and 3%, and 20% and 25% for the acalabrutinib and IdR/BR groups, respectively. Acalabrutinib, 100 mg twice daily, yielded an ORR of 94% in a phase 1b/2 study in 134 patients with relapsed/refractory CLL/SLL and a median of 2 prior therapies^44^. The ORR was not significantly affected by adverse genomic features. The estimated 45-month PFS rate was 62% (the median PFS had not been reached for the overall population but was 36 and 33 months for patients with del17p and complex karyotype, respectively). Diarrhea (52%) and headache (51%) were common; atrial fibrillation and major bleeding occurred in 7% and 5% of patients, respectively. Grade ≥3 neutropenia, pneumonia, hypertension, anemia, and diarrhea were observed in 5–14% of patients. Given its improved safety profile, acalabrutinib has been studied specifically in patients with CLL (n = 33) intolerant to ibrutinib^45^. The most common treatment-emergent AEs on acalabrutinib were diarrhea (58%), headache (39%), and cough (33%). Grade 3/4 neutropenia and thrombocytopenia occurred in 12% and 9%, respectively, although the overall rate of grade 3/4 AEs was 58%. A total of 72% of ibrutinib-related AEs did not recur on acalabrutinib, and 13% recurred at a lower grade. After a median follow-up of 19 months, 23 of the 33 patients remained on acalabrutinib. The ORR was 76%, and the median PFS and duration of response (DOR) to acalabrutinib had not been reached.

Zanubrutinib is another potent, second-generation BTK inhibitor with improved selectivity over ibrutinib that has been approved by the FDA for patients with MCL who have received ≥1 prior therapy. Administered twice daily like acalabrutinib, zanubrutinib has not been approved yet for patients with CLL but has demonstrated high efficacy with low toxicity in early phase trials^46^. Clinical trial results to date with zanubrutinib in patients with CLL/SLL are summarized in Table 1. A randomized, phase 3 trial (ALPINE) will compare zanubrutinib to ibrutinib in the relapsed/refractory setting^47^.

**Table 1.** Clinical trial results with zanubrutinib monotherapy in patients with CLL/SLL.

Phase	Eligibility	Patients	Efficacy	Safety	Reference
1 (FIH)	R/R B-cell malignancies in dose escalation portion (part 1); disease- specific cohorts in expansion portion (part 2)	n = 94; 22 TN, 72 R/R; median 2 prior therapies. Del17p/*TP53*^mut^, 19.1%; del11q, 23.3%; unmutated *IGHV*, 66.7%; LAD >10 cm, 5.3%	In 78 evaluable pts, ORR 96.2% (2.6% CR + 80.8% PR + 12.8% PR_L_); estimated 12 m PFS 100% at median f/u 13.7 m, median PFS not reached	No DLT observed; 160 mg bid selected as RP2D based on sustained >95% BTK occupancy in LN. No deaths. Gr 3/4 anemia, neutropenia, HTN, pneumonia in >1 patient	46
1/2 (update of above study with median f/u 29.5 m)	As above	n = 123; 22 TN, 101 R/R; median 2 prior therapies. Del17p, 16.2%; *TP53*^mut^, 31%; del11q, 23.5%; unmutated *IGHV*, 68.3%; LAD >5 cm, 38.2%	n = 123; ORR 100% (22.7% CR + 77.3% PR) in TN pts, 95% (13.9% CR + 72.3% PR + 7.9% PR_L_) in R/R pts. Median PFS 32.2 m for TN pts, 23.1 m for R/R pts	n = 123; gr ≥3 AEs in 61.8%, SAEs in 47.2%, AEs led to D/C in 4.1%. Most common AEs (>20%): contusion, URI, diarrhea, cough, headache, fatigue. Gr ≥3 a fib: 1.6%	52
3 (SEQUOIA)	Multiple cohorts (TN); cohort 1, Z vs. BR in non-del17p; cohort 2, Z alone in del17p; cohort 3, Z + V in del17p	Cohort 2 (Arm C) only: n = 109; all with del17p, TN; del11q in 33.9%, unmutated *IGHV* in 61.5%, β2m >3.5 g/dL in 78.6%, LAD ≥5 cm in 38.5%; median f/u 18.2 m	n = 109; ORR 94.5% (3.7% CR/CRi + 87.2% PR + 3.7% PR_L_); median PFS, DOR, and OS not reached	Most common AEs (>10%): contusion, URI, neutropenia, diarrhea, nausea, constipation, rash, back pain, cough, arthralgia, fatigue. A fib or flutter: 2.8%. AEs led to D/C in 3.7%. Gr ≥3 AEs in >2% of pts: neutropenia, pneumonia	53

Abbreviations: AE, adverse event; a fib, atrial fibrillation; BR, bendamustine plus rituximab; BTK, Bruton’s tyrosine kinase; CLL, chronic lymphocytic leukemia; CR/CRi, complete response with or without count recovery; D/C, discontinuation; DLT, dose-limiting toxicity; DOR, duration of response; FIH, first in human; f/u, follow-up; gr, grade; HTN, hypertension; LAD, lymphadenopathy; LN, lymph node; m, month; ORR, overall response rate; OS, overall survival; PFS, progression-free survival; PR, partial response; PR_L_, partial response with lymphocytosis; pts, patients; RP2D, recommended phase 2 dose; R/R, relapsed/refractory; SAE, serious adverse event; SLL, small lymphocytic lymphoma; TN, treatment-naive; tox, toxicity; URI, upper respiratory infection; V, venetoclax; Z, zanubrutinib.

As alluded to above, acquired mutations, e.g. C481S, in BTK (and gain-of-function mutations in phospholipase C gamma 2 [PLCG2] immediately downstream of BTK in the BCR signaling cascade) underlie most cases of resistance to irreversible BTK inhibitors, and their acquisition has been shown to precede clinical relapse or progression^48^. These observations have led to the development of third-generation BTK inhibitors.

## Third-generation BTK inhibitors

Third-generation BTK inhibitors were designed to circumvent the development of resistance to first- and second-generation BTK inhibitors due to point mutations at the C481 residue. This class of compounds, e.g. LOXO-305^49^, SNS-062 (vecabrutinib)^50^, and ARQ-531^51^, binds to an allosteric site of the kinase. These agents are in early clinical development in patients with relapsed/refractory CLL and MCL, primarily those who have failed therapy with ibrutinib. Results on 94 patients with CLL/SLL (median 4 prior therapies, including a BTK inhibitor in 84%, a PI3K inhibitor in 21%, and venetoclax in 31%) enrolled in the phase 1/2 BRUIN trial of LOXO-305 were recently presented^49^. Del17p was present in 21%, *TP53*^mut^ in 30%, and unmutated *IGHV* in 84%. The only treatment-emergent AEs observed in ≥10% of patients were fatigue (16%) and diarrhea (15%). The recommended phase 2 dose was 200 mg daily. A total of 65 patients were evaluable for efficacy; the ORR was 57% after a median follow-up of 3 months (77% among 26 patients with at least 6 months of follow-up). Responses were not influenced by the presence or absence of a pre-treatment BTK C481 mutation, reason for prior BTK inhibitor discontinuation (i.e. resistance or intolerance), or other classes of prior therapy received.

## Venetoclax

The BH3-mimetic venetoclax has quickly gone from its first FDA approval in 2016 for patients with relapsed, del17p CLL^54^ to full approval (in combination with CD20 monoclonal antibodies) for all CLL patients. The issue of tumor lysis syndrome (TLS) has been successfully mitigated by the implementation of a dose ramp-up, in which the venetoclax dose is raised in a step-wise fashion every week from 20 mg to 50 mg to 100 mg to 200 mg to the target dose of 400 mg daily^55^. Importantly, venetoclax is able to eradicate blood and marrow MRD even as a single agent, an effect not seen with ibrutinib.

In an update of the original pivotal trial in 158 patients with (mostly) relapsed/refractory CLL with del17p and a median of 2 prior therapies, 71% of whom had *TP53*^mut^ and 48% of whom had nodes ≥5 cm, a blood MRD clearance rate of 30% was reported^56^. Median time on venetoclax was 23.1 months. The investigator-assessed ORR was 77% and the estimated 2-year PFS was 54% (63% and 50%, respectively, in the 16 patients who had received prior kinase inhibitors).

Pooled data from several early phase studies of venetoclax in patients with relapsed/refractory CLL were analyzed to comprehensively characterize the safety profile of venetoclax monotherapy as well as identify factors predictive of efficacy. Among 350 patients who had received a median of 3 prior therapies (including ibrutinib or idelalisib in 42%), the median duration of exposure to venetoclax was 16 months^57^. The most common AEs were diarrhea, neutropenia, nausea (frequency for each ≈40%), anemia, fatigue, and URI (each in the 25–30% range). The most common grade 3/4 AEs were cytopenias, particularly neutropenia (neutrophils are dependent upon the pro-survival function of BCL-2). Serious infections occurred in 15%. In a similar analysis of efficacy (n = 436), the ORR was 75%, including a 22% CR/CRi rate^58^. Undetectable MRD in the blood and marrow, respectively, was achieved by 27% and 16% of the patients. The estimated PFS was 30.2 months. For those who achieved CR/CRi, the 3-year PFS estimate was 83%. Achievement of CR/CRi or undetectable MRD predicted for longer DOR, while bulky lymphadenopathy (≥5 cm) and refractoriness to prior BCR inhibitor therapy were significantly associated with lower CR rates and shorter DOR. Del17p and/or *TP53*^mut^ and *NOTCH1*^mut^ were consistently associated with shorter DOR, but not ORR. Fewer prior therapies predicted for higher CR rates, but not DOR. Of practical importance, the efficacy of single agent venetoclax after failure/discontinuation of BCR pathway inhibitors has been specifically studied; these data are summarized in Table 2.

**Table 2.** Efficacy of venetoclax monotherapy after ibrutinib, idelalisib, or both.

Study design	Patients	Efficacy	Safety	Reference
Open-label, non- randomized, multi- center, phase 2 trial (prior ibrutinib cohort)	91 pts with R/R CLL who had received ibr as their last BCRi; median f/u 14 m. Ibr D/Ced due to disease progression in 55% and AEs in 33%; 68% refractory to ibr, 31% relapsed on or after ibr D/C	ORR 65% (9% CR/CRi + 3% nPR + 52% PR). Median PFS 24.7 m, median OS not reached. Estimated 12-m PFS, 75%; estimated 12-m OS, 91%	Most common treatment-emergent gr 3/4 AEs: neutropenia (51%), thrombocytopenia (29%), anemia (29%), leukopenia (19%), lymphopenia (15%)	61
As above (prior idelalisib cohort)	36 pts with R/R CLL who had received ide as their last BCRi; median duration of prior ide 9 m; reason for ide D/C: tox with subsequent PD in 61% and PD on ide in 36%; median f/u 14 m	ORR 67% (8% CR/CRi + 58% PR). Median PFS and OS not reached. Estimated 12-m PFS, 79%; estimated 12-m OS, 94%	Most common gr 3/4 AEs: neutropenia (50%), thrombocytopenia (25%), anemia (17%); all grades: neutropenia (56%), diarrhea (42%), URI (39%), thrombocytopenia (36%), nausea (31%), fatigue (28%), cough (22%), rash (22%), anemia (22%)	62
As above (post hoc analysis of pts who had received >1 prior BCRi)	28 pts with R/R CLL who had received >1 BCRi (ibr and ide in 86%); median f/u 11.8 m	ORR 43% (4% CR + 39% PR); median PFS 16.4 m. Estimated 12-m PFS, 58%; estimated 12-m OS, 89%	Common AEs: gr 1/2 GI tox and gr 3/4 cytopenias	63

*Abbreviations:* AE, adverse event; BCRi, B-cell receptor pathway inhibitor; CLL, chronic lymphocytic leukemia; CR/CRi, complete response with or without count recovery; D/C, discontinuation; f/u, follow-up; GI, gastrointestinal; gr, grade; ibr, ibrutinib; ide, idelalisib; m, month; nPR, nodular partial response; ORR, overall response rate; OS, overall survival; PFS, progression-free survival; PR, partial response; pts, patients; R/R, relapsed/refractory; tox, toxicity; URI, upper respiratory infection.

## Venetoclax combinations with CD20 monoclonal antibodies: finite duration regimens

### Venetoclax with rituximab

MURANO was a phase 3 RCT that compared the combination of venetoclax plus rituximab (Ven-R, venetoclax for 2 years and rituximab for 6 cycles) to 6 cycles of BR in 389 patients with relapsed/refractory CLL (1–3 prior therapies)^59^. Del17p was detected in 26.9% of 342 patients tested, *TP53*^mut^ in 26.3% of 376 tested, and unmutated *IGHV* in 68.3% of 360 patients tested. After a median follow-up of 23.8 months, the 2-year (investigator-assessed, verified by independent review) PFS rate was 84.9% for Ven-R and 36.3% for BR, findings that led to the FDA approval of the Ven-R regimen for patients with relapsed/refractory CLL. The 2-year PFS rates for Ven-R were very similar in patients with and without del17p (81.5% and 85.9%, respectively), while those for BR were 27.8% and 41%, respectively. The ORR and CR/CRi rates were 92.3% and 8.2%, respectively, in the Ven-R group, and 72.3% and 3.6% in the BR group. OS at 2 years favored Ven-R as well (91.9% versus 86.6% with BR). Rates of MRD clearance in both blood (available in 94.1%) and marrow (available in 29.6%) were much higher in the Ven-R group (62.4% at 9 months and 83.5% at any time in the peripheral blood) than in the BR group (13.3% at 9 months and 23.1% at any time), and MRD clearance rates at the 9-month time point predicted subsequent PFS. Grade 3/4 neutropenia occurred more frequently in the Ven-R group, but the rates of grade 3/4 febrile neutropenia (FN) and infections were higher with BR. Grade 3/4 TLS occurred in 3.1% of patients in the Ven-R group.

In the most recent update of this trial (median follow-up, 59.2 months), the median PFS was 53.6 months for Ven-R and 17 months for BR, and the 5-year OS estimates were 82.1% and 62.2% for Ven-R and BR, respectively^60^. For patients completing the 2 years of venetoclax, the PFS estimate 36 months after the end of treatment (EOT) was ≈51.1.%. For Ven-R patients who reached EOT without disease progression, undetectable MRD at EOT (83/118) predicted improved OS (3-year post-EOT survival estimate 95.3% versus 85% for those [35/118] with detectable MRD at EOT). Of the 83 Ven-R patients with undetectable MRD at EOT, 32 remained MRD negative at 5 years, 4 had disease progression without confirmation of MRD conversion, and 47 developed MRD conversion a median of 19.4 months after EOT; of these 47, 19 subsequently developed progressive disease after another 25.2 months (median). Baseline risk factors associated with increased risk of MRD conversion among patients with undetectable MRD at EOT were del17p, complex karyotype, and unmutated *IGHV*. No new safety signals were identified. Importantly, peripheral blood MRD assessment has been shown to be a good surrogate for bone marrow MRD assessment in the context of venetoclax therapy in relapsed/refractory CLL, correlating equally well with long-term outcomes^64^. Furthermore, no new attainment of MRD-negative status was observed after 24 months; concurrent rituximab hastened the attainment of undetectable MRD, while complex karyotype was associated with lower rates of undetectable MRD at 12 months. Of interest, a retrospective, real-world analysis (n = 321) found no differences in terms of ORR, PFS, and OS between heavily pre-treated CLL patients receiving venetoclax monotherapy and those receiving venetoclax plus an anti-CD20 monoclonal antibody after a median follow-up of 13.4 months^65^.

### Venetoclax with obinutuzumab

In a phase 1b study in 32 patients with previously untreated CLL and 50 with relapsed/refractory CLL, venetoclax plus obinutuzumab (Ven-G), administered for 6 cycles followed by venetoclax to complete 1 year (for treatment-naïve patients) or until disease progression (in relapsed/refractory patients), yielded ORRs of 95% (37% CR/CRi) and 100% (78% CR/CRi) in the relapsed/refractory and frontline cohorts, respectively^66^. MRD was undetectable in the peripheral blood ≥3 months after the last obinutuzumab dose in 64% of the relapsed/refractory patients and 91% of the previously untreated patients.

The pivotal German CLL14 phase 3 RCT that led to the approval of Ven-G in the frontline setting compared this regimen 1:1 to the combination of chlorambucil and obinutuzumab in 432 treatment-naïve patients with CLL and a CIRS score >6 or a creatinine clearance <70 mL/minute^67^. The median age was 72, 13.8% of the patients had *TP53*^mut^, deletion or both, and 59.8% had unmutated *IGHV*. Obinutuzumab was administered for 6 cycles, while both venetoclax and chlorambucil (0.5 mg/kg on days 1 and 15 of each 28-day cycle) were administered for 12 cycles. Venetoclax was initiated on day 22 of cycle 1 in order to mitigate TLS risk. No crossover was permitted. After a median follow-up of 28.1 months, the estimated 2-year PFS rate was 88.2% in the Ven-G group and 64.1% in the chlorambucil/obinutuzumab group, a statistically significant benefit observed across pre-specified subgroups. Both the ORR and the CR rate were significantly higher in the Ven-G arm (ORR 84.7% versus 71.3% and CR 49.5% versus 23.1%, *P* <0.001 for both comparisons). OS was not significantly different between the groups (median not reached in either group). MRD clearance in the peripheral blood (75.5% versus 35.2%, *P* <0.001) and bone marrow (56.9% versus 17.1%, *P* <0.001) 3 months after therapy completion was also significantly better with Ven-G than with chlorambucil/obinutuzumab. In both arms, undetectable MRD in the peripheral blood at EOT correlated with favorable 24-month PFS rates in landmark analyses^68^. Most AEs occurred at similar frequencies in the two treatment arms. Neutropenia was the most common grade 3/4 AE, while grade 3/4 FN occurred in 5.2% (all grades, 17.5%) and 3.7% (all grades, 15%) of the Ven-G and chlorambucil/obinutuzumab patients, respectively. No cases of clinical TLS^69^ were observed in either arm. A total of 56% of Ven-G patients with undetectable MRD at EOT had already achieved this after the combination phase of treatment, while in 25%, the MRD response deepened during the 6 cycles of venetoclax monotherapy following the combination phase^70^. In a landmark analysis of PFS after EOT, Ven-G patients with MRD levels ≤10^-5^ had a 2-year PFS rate of ≈93%, while those with MRD levels >10^-2^ had a 2-year PFS rate of ≈37%. After a median follow-up of 39.6 months (all patients off treatment for at least 24 months), median PFS had not been reached in the Ven-G group versus 35.6 months in the chlorambucil/obinutuzumab group^71^.

## Ibrutinib plus venetoclax

The combination of ibrutinib with venetoclax is a logical one given preclinical evidence of synergism^72,73^, the different disease compartments in which each drug appears most active (lymph nodes for ibrutinib, blood and marrow for venetoclax), and their high clinical efficacy as monotherapy. A number of clinical trials have evaluated this combination in both newly diagnosed and relapsed/refractory CLL.

In the MDACC phase 2 trial in 80 previously untreated high-risk/older patients, 75 of whom received the combination, venetoclax was introduced after 12 weeks (3 cycles) of ibrutinib monotherapy and administered for 24 cycles, following which ibrutinib could continue depending on bone marrow MRD status at the end of combination treatment^74^. After 12 cycles of combination therapy, 88% of patients had achieved CR/CRi, and 61% had undetectable bone marrow MRD. Responses improved and deepened with time and were seen independent of *IGHV* mutation status, fluorescence in situ hybridization (FISH) category, and *TP53/NOTCH1/SF3B1* mutation status. Atrial fibrillation developed in 12 patients (15%) and was the most common reason for dose reduction of ibrutinib. FN occurred in four patients; neutropenia was the most common reason for dose reduction of venetoclax. In the most recent update (median follow-up, 33.8 months) of this trial, the rate of undetectable bone marrow MRD after 24 cycles of combination therapy by intention-to-treat (ITT) analysis was 66%, and that at any time point was 75%^75^. A total of two patients developed RT; no patient had progression of their CLL. Of 24 patients who had detectable bone marrow MRD after 12 cycles of combination therapy, 12 achieved undetectable MRD in the bone marrow by the end of cycle 24. Based on this observation, the study has been amended to allow a further 12 cycles of combination therapy in patients with detectable bone marrow MRD at the end of cycle 24.

The “MRD cohort” of the industry-sponsored, CAPTIVATE phase 2 study (n = 164) was very similarly designed, with MRD status evaluated in the peripheral blood after 6, 9, and 12 cycles and in the bone marrow after 12 cycles of combination ibrutinib and venetoclax (following a 3-cycle ibrutinib lead-in)^76^. The median age was 58, and the proportions of patients with del17p, del11q, del17p or *TP53*^mut^, complex karyotype, and unmutated *IGHV* were 16%, 17%, 20%, 19%, and 60%, respectively. A total of 86 patients achieved confirmed undetectable MRD serially over ≥3 cycles, in both blood and marrow, after 12 cycles of combination therapy, and were randomized 1:1 to receive placebo or ibrutinib monotherapy; 1-year disease-free survival (DFS) did not differ significantly between these arms. A total of 63 patients did not achieve undetectable MRD as defined above and were randomized to continue ibrutinib plus venetoclax or ibrutinib alone. Rates of undetectable MRD in the blood and marrow improved in these patients. Rates of 30-month PFS were >95% across all four arms.

A total of 80 patients were also enrolled to the relapsed/refractory cohort of the MDACC trial^77^. The median follow-up was 27 months when last presented, and 74 patients received combination treatment. By ITT analysis, the rates of undetectable bone marrow MRD after 12 cycles of combination therapy and at any time point (best response) were 40% and 56%, respectively. When considering evaluable patients only, this rate was 47% after 12 cycles and 68% after 24 cycles of combination treatment. A total of 2 patients developed CLL progression after 24 cycles of combination therapy, and 1 developed RT while on combination therapy. Grade 3/4 neutropenia occurred in 43% and atrial fibrillation in 8%. The doses of ibrutinib and venetoclax were reduced in 57% and 35% of patients, respectively.

The UK CLARITY phase 2 study also evaluated the combination of ibrutinib (administered alone for the first 8 weeks) and venetoclax in 50 patients with relapsed/refractory CLL^78^. Patients had received 1–6 prior therapies (median 1), 20% had del17p, 25% del11q, and 75% unmutated *IGHV*^79^. Duration of therapy was based on peripheral blood and bone marrow assessments after 6, 12, and 24 cycles of combination treatment. Venetoclax was administered for a maximum of 24 cycles, but both drugs could be stopped after 12 cycles of combination therapy if both blood and marrow MRD negativity were attained after 6 cycles of combination therapy. The ORR was 89% (n = 49) after 12 cycles of combination therapy, including CR/CRi in 51% and PR in 38%. As in the MDACC trial, responses deepened over time, with the rate of undetectable MRD in the bone marrow increasing from 40% after 12 cycles of combination treatment to 48% after 24 cycles.

The UK CLARITY phase 2 study also evaluated the combination of ibrutinib (administered alone for the first 8 weeks) and venetoclax in 50 patients with relapsed/refractory CLL^78^. Patients had received 1–6 prior therapies (median 1), 20% had del17p, 25% del11q, and 75% unmutated *IGHV*^79^. Duration of therapy was based on peripheral blood and bone marrow assessments after 6, 12, and 24 cycles of combination treatment. Venetoclax was administered for a maximum of 24 cycles, but both drugs could be stopped after 12 cycles of combination therapy if both blood and marrow MRD negativity were attained after 6 cycles of combination therapy. The ORR was 89% (n = 49) after 12 cycles of combination therapy, including CR/CRi in 51% and PR in 38%. As in the MDACC trial, responses deepened over time, with the rate of undetectable MRD in the bone marrow increasing from 40% after 12 cycles of combination treatment to 48% after 24 cycles.

Another approach being pursued by investigators at MDACC is the addition of venetoclax as “consolidation” in high-risk patients already on ibrutinib for ≥1 year and responding, but with detectable disease. The maximum duration of combination therapy is 2 years. Venetoclax is stopped after two consecutive undetectable MRD assessments in the bone marrow; ibrutinib can continue. Updated results from this study (on 45 patients) were recently presented^80^. A total of two patients (4%) were in CR at venetoclax initiation, and 45% after 1 year. After 6 and 12 months of combination therapy, 40% (17/42) and 64% (21/33), respectively, had achieved bone marrow MRD clearance. Treatment has been well tolerated. Only one patient had CLL progression after 18 months of combination therapy.

## Triple targeted therapy regimens

Given the successes of ibrutinib plus venetoclax regimens^74,78^ and of each of these drugs individually when combined with obinutuzumab^19,67^, the advent of “triple” regimens incorporating a BTK inhibitor, venetoclax, and obinutuzumab was a logical next step. The group at OSU studied the combination of ibrutinib, venetoclax, and obinutuzumab given for a finite duration in patients with both treatment-naïve (n = 25) and relapsed/refractory CLL (n = 25)^81^. Initiation of the 3 agents was staggered so that obinutuzumab was administered in cycles 1 through 8, ibrutinib in cycles 2 through 14, and venetoclax in cycles 3 through 14^82^. Infusion reactions (66%, all grades 1/2), hypertension (70%, 32% grade 3/4), neutropenia (76%, 56% grade 3/4), and thrombocytopenia (80%, 34% grade 3/4) were extremely frequent. With 24.2 months of follow-up (median) of the treatment-naïve cohort and 21.5 months (median) for the relapsed/refractory cohort, the ORRs at EOT were 84% and 88%, respectively. MRD eradication in both blood and marrow at EOT occurred in 67% and 50% of patients, respectively, in the treatment-naïve and relapsed/refractory groups, while 28% of patients were in MRD-negative (blood and marrow) CR at EOT in each cohort. Interestingly, in this trial, the ORRs mid-therapy (i.e. at the end of cycle 8) were higher than those at EOT in both treatment-naïve and relapsed/refractory patients. This was also true of the rate of MRD clearance in the relapsed/refractory cohort, but not in the treatment-naive cohort.

Investigators from the DFCI are testing the combination of acalabrutinib, venetoclax, and obinutuzumab (AVO) in the upfront setting (n = 44)^83^. Baseline characteristics as recently presented include a median age of 63, *TP53* aberrations in 39%, del11q in 27%, complex karyotype in 20%, and unmutated *IGHV* in 66% of patients. In this study, acalabrutinib commences on cycle 1, day 1, and obinutuzumab (for 6 cycles) begins in cycle 2, while venetoclax is initiated in cycle 4 and continued for 12 cycles. Patients can discontinue all therapy if MRD negative in the bone marrow after 15 cycles; those not achieving this (primary endpoint) continue acalabrutinib and venetoclax to complete 24 cycles. The ORR was 97% even before the initiation of venetoclax and improved further to 100% (43% CR/CRi) at later time points. After 15 cycles, by ITT analysis, 78% had achieved undetectable MRD in the bone marrow (31% CR with undetectable bone marrow MRD) and 84% in the peripheral blood. All 10 patients with *TP53* aberrations who completed 15 cycles responded, and responses did not differ by *IGHV* mutation status. No patients had disease progression after a median follow-up of 19 months, and 11 discontinued therapy per protocol after attaining undetectable bone marrow MRD after 15 cycles. A similar combination trial (NCT04169737) has been initiated at MDACC in which patients with either relapsed/refractory or high-risk, previously untreated CLL/SLL are randomly assigned to receive either early or late obinutuzumab (all patients receive acalabrutinib and venetoclax) to address the role of CD20 targeting in this context.

The combination of zanubrutinib, obinutuzumab, and venetoclax, the so-called BOVen regimen, has also been studied in the frontline setting (n = 39)^84^. A total of 72% of patients had unmutated *IGHV*, and 15.4% had del17p or *TP53*^mut^. Both zanubrutinib and obinutuzumab began in cycle 1, obinutuzumab was given for 8 cycles, and venetoclax was initiated in cycle 3. At a median follow-up of 14+ months, the rates of undetectable MRD in the bone marrow (primary endpoint) and peripheral blood were 84% and 92%, respectively. A total of 29 patients (77%) were able to discontinue therapy per protocol, having achieved undetectable MRD in both blood (2 consecutive samples) and marrow.

## Venetoclax resistance and salvaging patients who fail venetoclax

While all the underlying mechanisms of resistance to venetoclax in CLL have yet to be elucidated, the discovery that acquisition of the G101V mutation in BCL-2, greatly reducing its affinity for venetoclax, could pre-date and lead to clinical disease progression in some patients was an important breakthrough^85^. These investigators later reported the co-occurrence of multiple other BCL-2 mutations, e.g. at Asp103, along with G101V in the context of disease progression on venetoclax^86^. Others have reported clonal evolution on venetoclax therapy, including mutations in *BTG1* and homozygous deletions of *CDKN2A/B*, as well as *BRAF* mutations and amplification of *PD-L1* as possible mechanisms^87^. Mcl-1 overexpression and mitochondrial reprogramming, involving regulators of transcription and cellular energy metabolism, have also been implicated as driving venetoclax resistance in lymphoid malignancies^88^.

With the increasing use of ibrutinib or acalabrutinib and venetoclax earlier in therapy, a scenario one is increasingly likely to encounter is that of patients who have failed both BTK and BCL-2 inhibition. Treatment options are very limited in this situation, an important area of unmet medical need. The results of chimeric antigen receptor-modified T-cell (CAR T-cell) therapy in CLL were underwhelming after the initial enthusiasm surrounding it^89^, and no CAR T-cell product is approved at present for patients with CLL; however, data presented at the 2020 ASH annual meeting on the investigational CD19-directed CAR T-cell product lisocabtagene maraleucel (liso-cel) appear promising, with a best ORR of 82% (CR/CRi in 45%) and rapid clearance of both blood and marrow MRD in most patients^90^. The patient population (n = 23) studied was a heavily pre-treated one (median 6 prior therapies), with all patients having received prior ibrutinib and over half having received venetoclax as well. The results with this product in combination with ibrutinib in a very similar patient population (n = 19) have been similarly encouraging^91^. Albeit early, anti-CD19 CAR NK (natural killer)-cells derived from cord blood have shown efficacy in patients with relapsed/refractory non-Hodgkin’s lymphoma or CLL without cytokine release syndrome, neurotoxicity, or graft-versus-host disease^92^.

An important multi-center, retrospective study identified 326 patients with CLL who discontinued venetoclax (96% in the relapsed/refractory setting)^93^. Prior to venetoclax initiation, 82% of the patients had unmutated *IGHV*, 47% del17p, 45% *TP53*^mut^, 39% complex karyotype, 23% *BTK*^mut^, and 10% *PLCG2*^mut^. The median number of therapies prior to venetoclax was 3. Progressive CLL or RT was the reason for discontinuation of venetoclax in 50%. BTK inhibitor therapy following venetoclax discontinuation was highly effective in BTK inhibitor-naïve patients (ORR 84%; estimated median PFS 32 months). The ORR to BTK inhibition was 54% in BTK inhibitor-exposed patients, with the median PFS ranging from not reached in BTK inhibitor-intolerant patients to 4 months in resistant patients. Responses to PI3K inhibitor therapy (ORR 47%) were not durable (median PFS 5 months), even though all patients were PI3K inhibitor naïve. A total of 18 patients received CAR T-cell therapy and 66% responded (median PFS 9 months), while median PFS was not reached for the 19 patients who underwent allogeneic hematopoietic cell transplantation (allo-HCT).

## Conclusions

The BTK inhibitors and venetoclax have revolutionized the treatment of CLL, with “cure” or long-term treatment-free remission now seeming a realistic goal, although RT remains a formidable therapeutic challenge. Acalabrutinib and zanubrutinib promise to make BTK inhibition safer while preserving the efficacy of ibrutinib, at least with the limited follow-up available to date. Reversible BTK inhibitors will hopefully address the problem of resistance to ibrutinib and acalabrutinib. Equally, the appeal of “time-limited”, venetoclax-based therapy is obvious. The selection of upfront therapy needs to be tailored to the individual patient, as does the optimal sequencing of therapies. The ideal duration of “time-limited” therapy continues to be debated. After idelalisib, duvelisib was another PI3K inhibitor to receive FDA approval for relapsed/refractory CLL based on the demonstration of superior PFS versus ofatumumab in the DUO trial^94^, but the immune-mediated toxicities of this class of agents have precluded their widespread use. Nevertheless, development of umbralisib, a PI3K inhibitor with an apparently cleaner safety profile, in combination with ublituximab, a CD20 monoclonal antibody from the same company, continues, and positive results from the pivotal UNITY-CLL trial were recently presented (Table 3)^95,96^. In general, combination strategies have resulted in high rates of CR with undetectable MRD. CAR T-cells and CAR NK-cells appear promising as well and will likely be best positioned after failure of BTK inhibitor(s) and venetoclax.

**Table 3.** Results with the “U2” (umbralisib + ublituximab) regimen in relapsed/refractory CLL.

Study design	Regimen	Patients	Efficacy	Safety	Reference
Phase 1/1b, multi-center trial in pts with R/R B-NHL or CLL with dose escalation and expansion portions; TN CLL allowed in phase 1b	Umbralisib daily PO continuously; ublituximab IV on d1, 8, 15 of C1, d1 of C2–C6, and then d1 every 3 cycles (C7–C12)	n = 75 (22 pts with CLL/SLL, 53 with B-NHL). 9 CLL/SLL pts with del17p, 6 BTKi-exposed; median 2 prior therapies (0–7)	n = 21. ORR 62% (10% CR + 52% PR); ORR 40% in BTKi-exposed pts. Median DOR 25.89 m; median PFS 27.57 m. Median treatment duration 11 m. RP2D: 900 mg ublituximab and 800 mg micronized umbralisib	n = 75. MTD not reached; 1 DLT (gr 4 neutropenia) in a CLL/SLL pt. Gr 3/4 AEs: diarrhea (8%), neutropenia (28%, 50% in CLL/SLL pts), pneumonia (8%), abd pain (7%), transaminitis (4%). 2 cases of pneumonitis, 1 bx-proven colitis (median f/u 7.4 m)	95
Phase 3 trial of U2 versus obin-clb in pts with TN or R/R CLL	Umbralisib 800 mg daily PO continuously; ublituximab 900 mg IV on d1/2, 8, 15 of C1, d1 of C2–C6, and then d1 every 3 cycles. Obin 1,000 mg IV on d1/2, 8, 15 of C1, d1 of C2–C6. Clb 0.5 mg/kg PO on d1 and d15 of C1–C6	n = 421 (210 U2 + 211 obin-clb). Median age 67, 240 TN, 181 R/R (median 1 prior therapy). 10% with del17p, 20% del11q, 56% *IGHV* unmutated	Median PFS 31.9 m with U2 (38.5 m for TN and 19.5 m for R/R) vs. 17.9 m with obin-clb (26.1 m for TN and 12.9 m for R/R) after median f/u of 36.2 m. 24-m PFS rates 60.8% with U2 and 40.4% with obin-clb. ORR 83.3% with U2 vs. 68.7% with obin-clb	Gr 3/4 AEs of interest (U2 vs. obin-clb): neutropenia (30.6% vs. 34.7%), thrombocytopenia (3.4% vs. 13.1%), diarrhea (12.1% vs. 2.5%), IRR (1.9% vs. 3.5%), elevated liver enzymes (8.3% vs. 2%), colitis (3.4% vs. 0%), and pneumonitis (2.9% vs. 0%)	96

*Abbreviations:* abd, abdominal; AE, adverse event; B-NHL, B-cell non-Hodgkin’s lymphoma; BTKi, Bruton’s tyrosine kinase inhibitor; bx, biopsy; C, cycle; clb, chlorambucil; CLL, chronic lymphocytic leukemia; CR, complete response;** d, day; DLT, dose-limiting toxicity; DOR, duration of response; f/u, follow-up; gr, grade; IRR, infusion-related reaction; IV, intravenous; MTD, maximum tolerated dose; obin, obinutuzumab; ORR, overall response rate; PFS, progression-free survival; PO, oral; PR, partial response; pts, patients; RP2D, recommended phase 2 dose; R/R, relapsed/refractory; SLL, small lymphocytic lymphoma; TN, treatment-naïve.
